# Supplementary material for: A redox switch regulates the assembly and anti-CRISPR activity of AcrIIC1
Source: Nat Commun. 2022 Nov 18;13:7071. doi: 10.1038/s41467-022-34551-8 (PMC9674691; doi:10.1038/s41467-022-34551-8)
Supplement: Supplementary file 1 — Supplementary Information [file 41467_2022_34551_MOESM1_ESM.pdf]

A redox switch regulates the assembly and anti-CRISPR activity of AcrIIC1

Yanan Zhao[1†], Jiaojiao Hu[2†], Shan-Shan Yang[3†], Jing Zhong[1], Jianping Liu[2], Shuo Wang[4], Yuzhuo Jiao[1], Fang Jiang[1], Ruiyang Zhai[1], Bingnan Ren[1], Hua Cong[1], Yuwei Zhu[4], Fengtong Han[1], Jixian Zhang[1], Yue Xu[1], Zhiwei Huang[4], Shengnan Zhang[2] &

Fan Yang[1*]

1. School of Life Science and Technology, Harbin Institute of Technology, Harbin 150080, China
2. Interdisciplinary Research Center on Biology and Chemistry, Shanghai institute of Organic Chemistry, Chinese Academy of Sciences, Shanghai 201210, China
3. State Key Laboratory of Urban Water Resource and Environment, School of Environment, Harbin Institute of Technology, Harbin 150090, China
4. Center for Life Sciences, School of Life Science and Technology, Harbin Institute of Technology, Harbin 150080, China

* Corresponding author:

Fan Yang: fanyang115@hit.edu.cn

† These authors contributed equally: Yanan Zhao, Jiaojiao Hu, Shan-Shan Yang

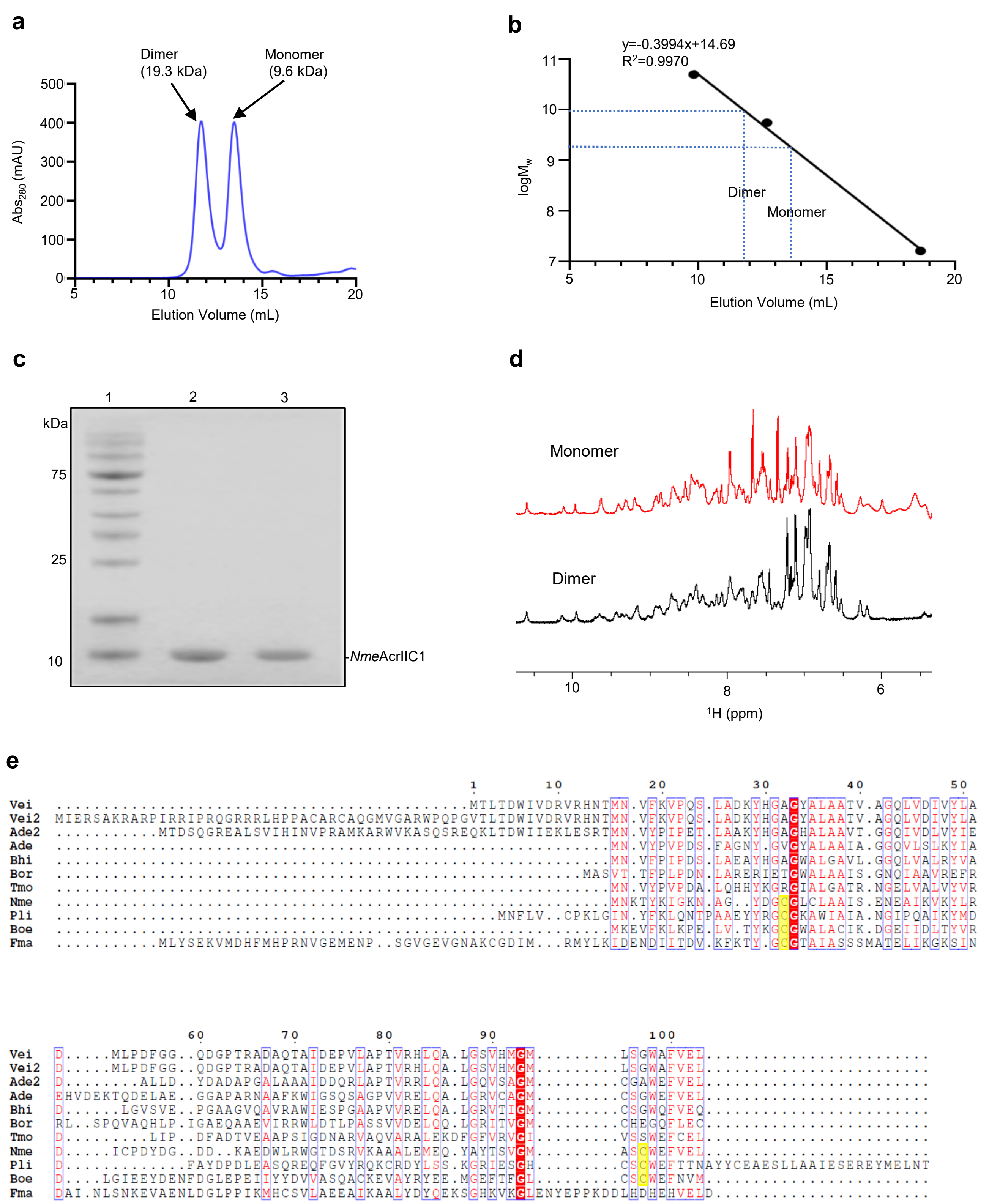

Supplementary Figure 1. Freshly purified *Nme*AcrIIC1 in the absence of reductant comprises two oligomeric states.

a SEC profile for the freshly purified *Nme*AcrIIC1 with non-reducing running buffer show two peaks corresponding to monomer and dimer, respectively. **b** The calibration curve for our SEC column (Superdex 75 Increase 10/300 GL (Cytiva)) using three globular protein standards. **c** SDS-PAGE gel indicates the two fractions from **a** are both 10 kDa. Lanes 2 and 3 are from the earlier and latter eluents, respectively. The experiment has been repeated more than three times with similar results. **d** 1D ^{1}H NMR spectra for the monomer (red) and the dimer (black). **e** Sequence alignment of *Nme*AcrIIC1 homologs, drawn with ESPript (http://espript.ibcp.fr/). Conserved cysteines at the same positions of Cys17 and Cys80 of *Nme*AcrIIC1 are highlighted in yellow. Source data are provided as a Source Data file.

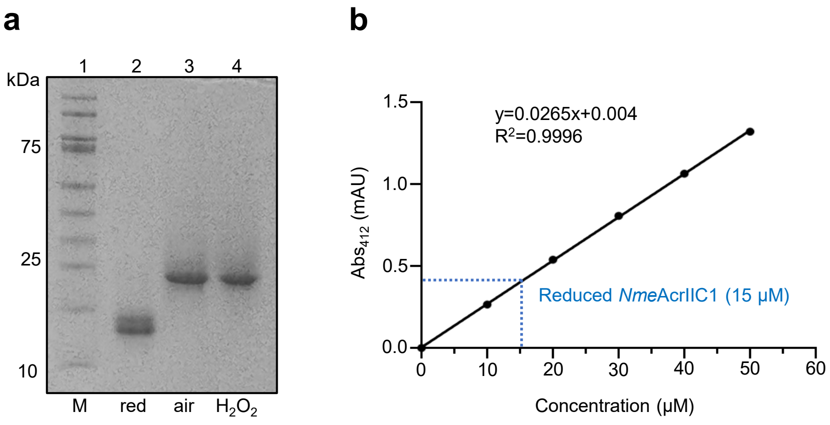

Supplementary Figure 2. Characterization of the oxidized *Nme*AcrIIC1 induced in two ways. **a** Native PAGE gel shows the bands of the reduced *Nme*AcrIIC1 (Lane 1), the oxidized *Nme*AcrIIC1 induced by the air (Lane 2), and the oxidized *Nme*AcrIIC1 induced with H_2O_2 (Lane 3). The experiment has been repeated more than three times with similar results. **b** Standard curve for DTT+DTNB mixture from Ellman's assays. The number of free sulfhydryl groups for the reduced *Nme*AcrIIC1 can be accurately extracted from the curve, while no absorbance at 412 nM can be detected for either form of oxidized *Nme*AcrIIC1. **c** Overlay of ^{1}H-^{15}N HSQCs for both forms of oxidized *Nme*AcrIIC1. Source data are provided as a Source Data file.

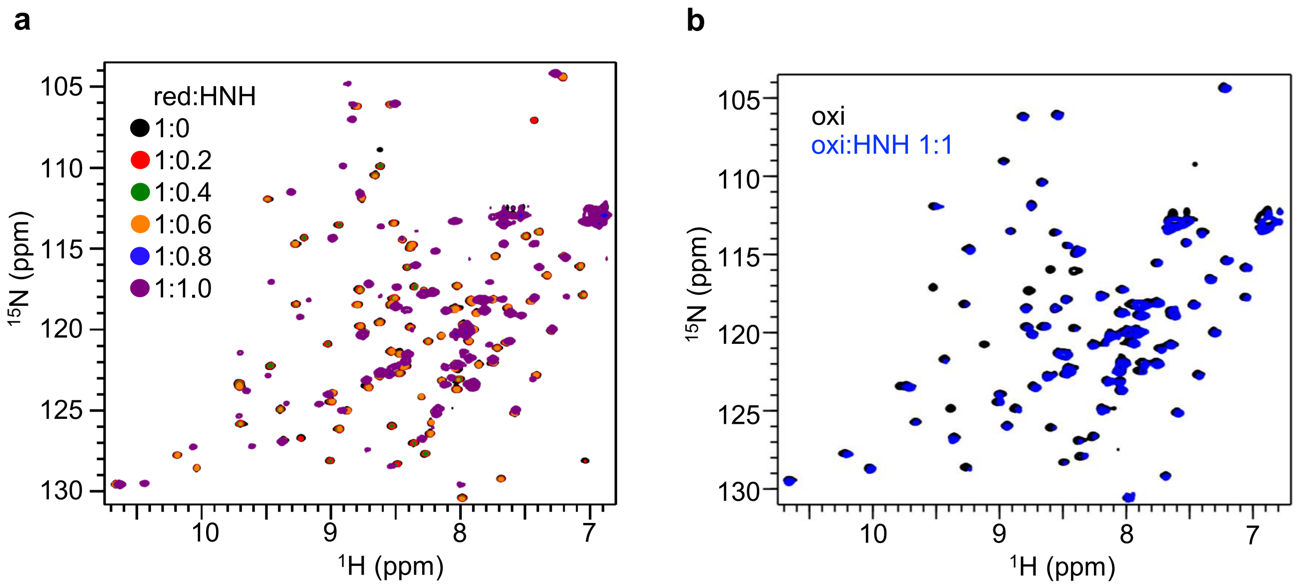

Supplementary Figure 3. Titrations of two states of *Nme*AcrIIC1 with *Nme*HNH by ^{1}H-^{15}N HSQCs.

a Superimposition of ^{1}H-^{15}N HSQC spectra for the reduced *Nme*AcrIIC1 bound to *Nme*HNH with the molar ratio of 1:0 (black), 1:0.2 (red), 1:0.4 (green), 1:0.6 (orange), 1:0.8 (blue), and 1:1 (purple). **b** Superimposition of ^{1}H-^{15}N HSQC spectra for the oxidized *Nme*AcrIIC1 in free form (black) and with 1:1 *Nme*HNH (blue).

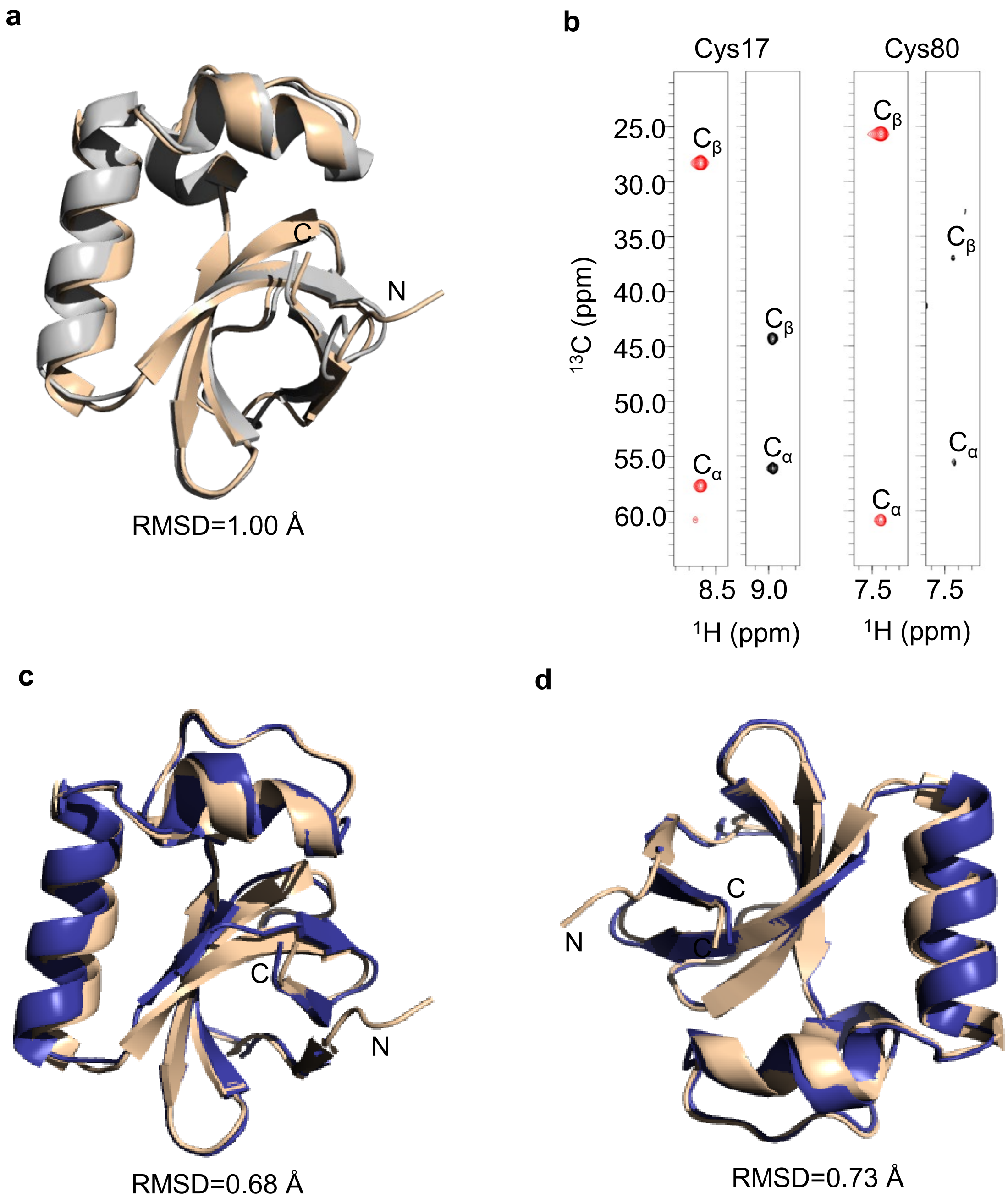

Supplementary Figure 4. Structural comparisons of different forms of *Nme*AcrIIC1.
a Overlay of *Nme*AcrIIC1s in its reduced form (wheat) and in complex with *Nme*HNH (gray). **b** CBCA(CO)NH strips showing C_α and C_β of Cys17 and Cys80 in their two redox forms (reduced form shown in red, oxidized form shown in black). **c** Overlay of *Nme*AcrIIC1s in its reduced form (wheat) and the subunit A of its oxidized form (blue). **d** Overlay of *Nme*AcrIIC1s in its reduced form (wheat) and the subunit B of its oxidized form (blue).

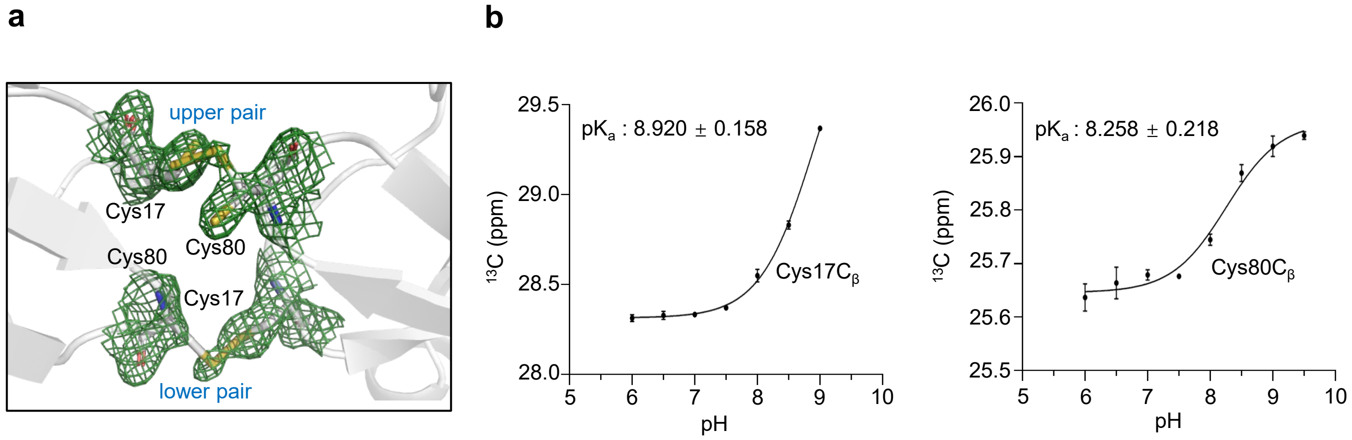

Supplementary Figure 5. The pair of Cys17 and Cys80 serves as a good redox switch.
a Close-up view of the Cys17-Cys80 disulfide bonds. 2Fo-Fc electron density map is contoured at 2.0 σ. The upper pair of Cys17-Cys80 adopts two redox conformations, either free cysteine or disulfide bonded. **b** pH titrations of C_β for Cys17 (left) and Cys80 (right) of *Nme*AcrIIC1, fitted using Eq.(2), which yield pKa values of 8.9 for Cys17 and 8.3 for Cys80. n=2 independent experiments have been performed. Dots represent mean, error bars represent SD. Source data are provided as a Source Data file.

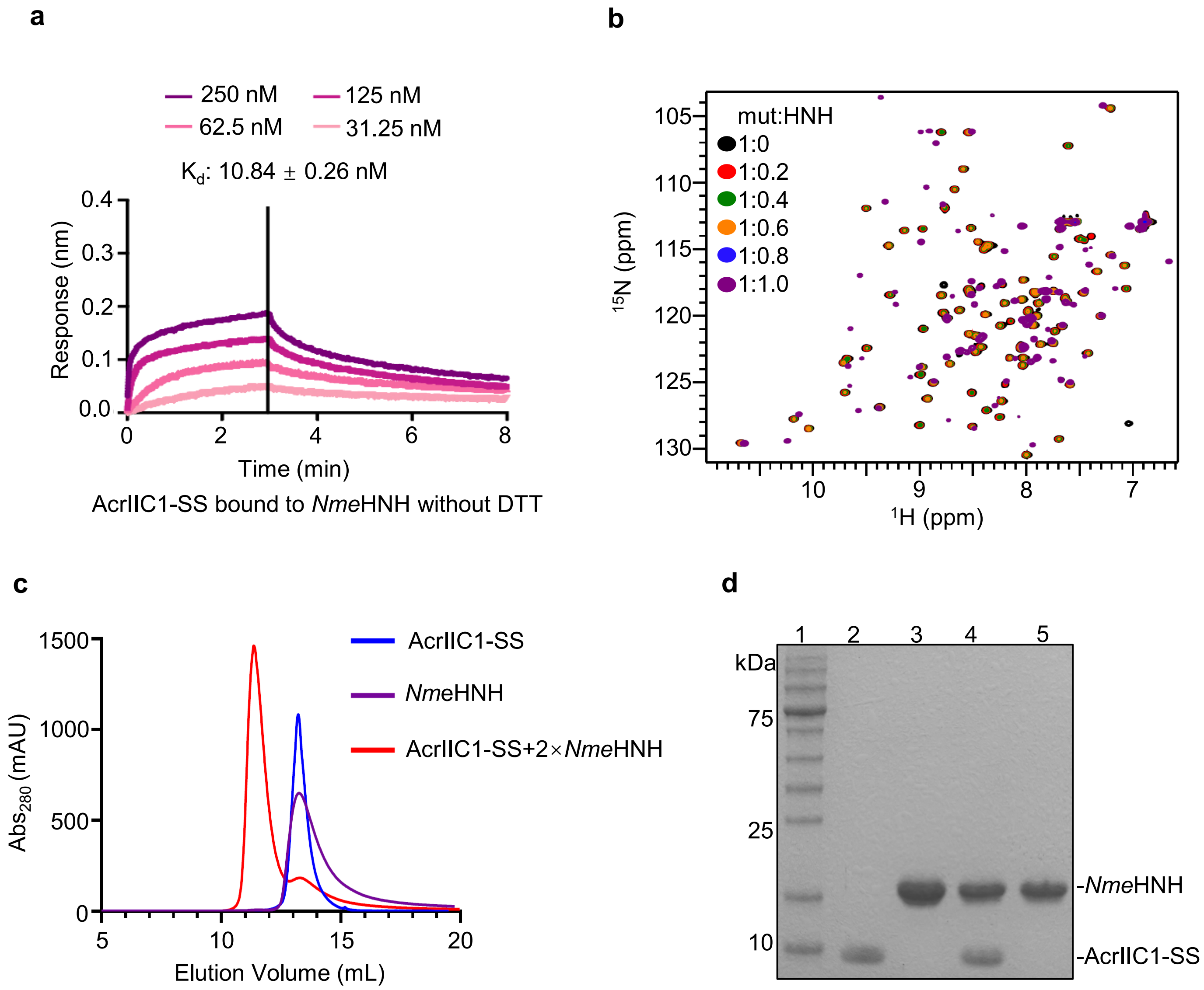

Supplementary Figure 6. Binding assays of AcrIIC1-SS to *Nme*HNH.

a K_d determined by BLI for AcrIIC1-SS bound to *Nme*HNH is 10.8 nM without reductant in the buffer. **b** Superimposition of ^{1}H-^{15}N HSQC spectra for AcrIIC1-SS bound to *Nme*HNH with the molar ratio of 1:0 (black), 1:0.2 (red), 1:0.4 (green), 1:0.6 (orange), 1:0.8 (blue), and 1:1 (purple). **c** SEC binding assay for AcrIIC1-SS bound to *Nme*HNH. **d** SDS-PAGE gel for the fractions from above runs (2, AcrIIC1-SS; 3, *Nme*HNH; 4, the complex eluent for AcrIIC1-SS bound to *Nme*HNH; 5, residual *Nme*HNH from the mixture of AcrIIC1-SS with *Nme*HNH). The experiment has been repeated more than three times with similar results. Source data are provided as a Source Data file.

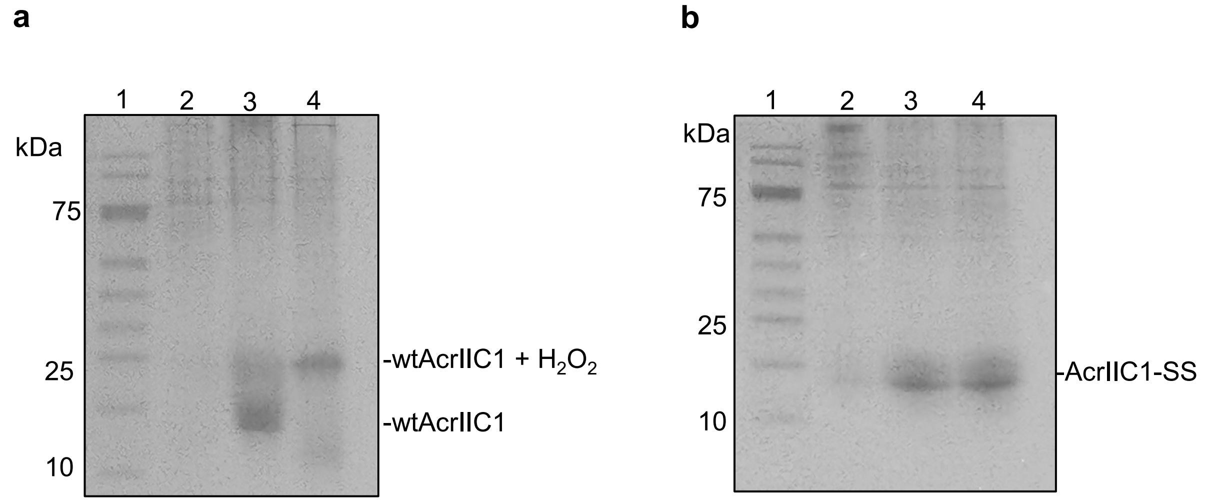

Supplementary Figure 7. Antimicrobial susceptibility assays accessing wtAcrIIC1 and AcrIIC1-SS activities *in vivo*.

a Native PAGE result shows the overexpression and oligomeric state of wtAcrIIC1 in cell (Lane 2, cell lysate before induction; Lane 3 and lane 5 correspond to cell lysate of panels 4 & 6 of **Fig. 6a**, respectively). **b** Native PAGE result shows the overexpression and oligomeric state of AcrIIC1-SS in cell (Lane 2, cell lysate before induction; Lane 3 and lane 5 correspond to cell lysate of panels 4 & 6 of **Fig. 6b**, respectively). The experiments have been repeated more than three times with similar results. Source data are provided as a Source Data file.

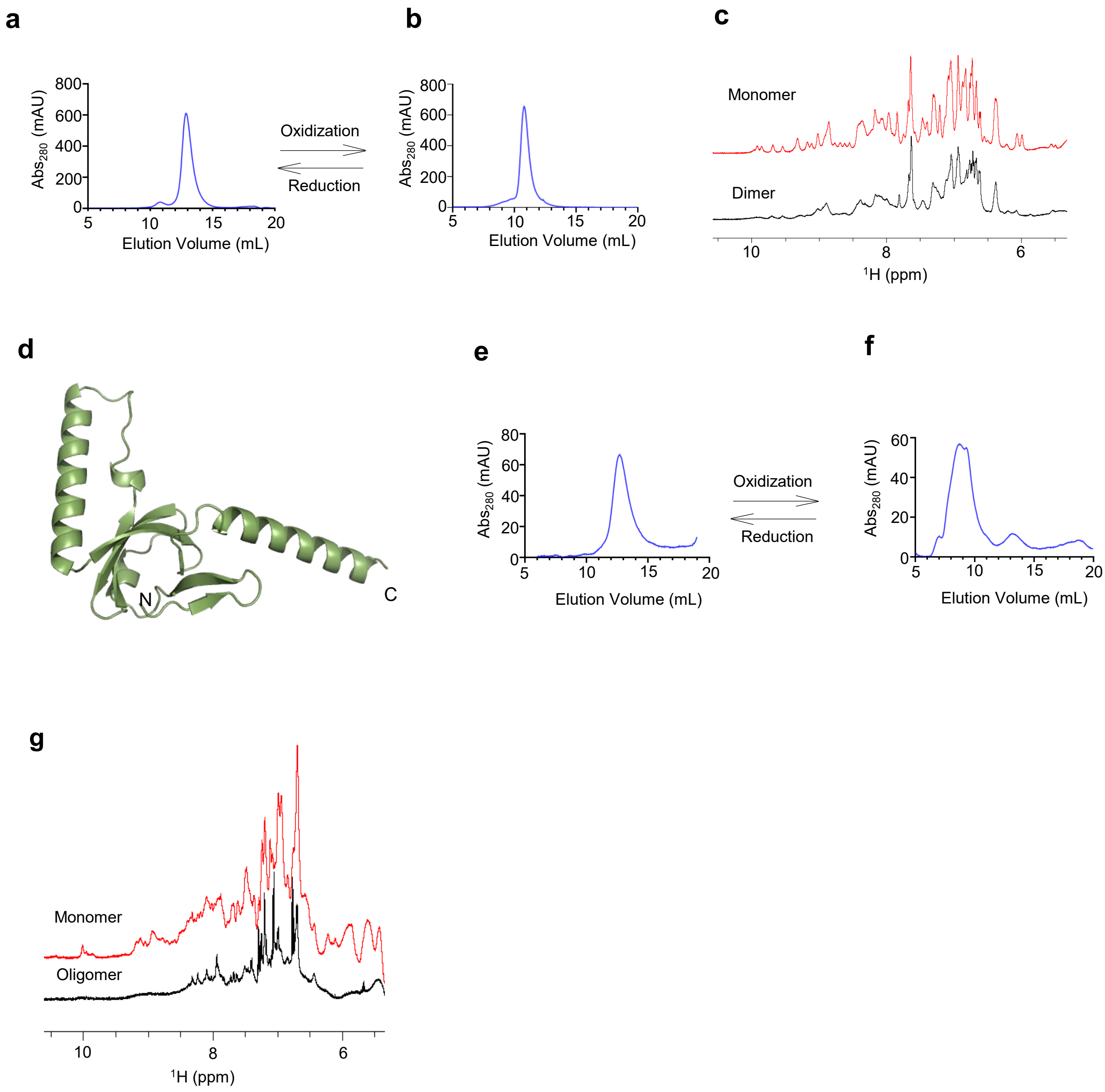

Supplementary Figure 8. Analysis of the redox-state transitions for *Boe*AcrIIC1 and *Pli*AcrIIC1.

a,b SEC runs show that the retention volume of *Boe*AcrIIC1 changes under different redox conditions, similar to the behavior of *Nme*AcrIIC1. **c**, 1D ^{1}H spectra show the difference between two redox states of *Boe*AcrIIC1. The spectrum for the reduced monomeric state is shown in red, and the one for the oxidized dimeric state is shown in black. **d** *Pli*AcrIIC1 structure modeled by AlphaFold. **e,f** SEC runs show that the retention volume of *Pli*AcrIIC1 changes under different redox conditions. The reduced state corresponds to the monomer, and the oxidized state corresponds to the oligomer. **g** 1D ^{1}H spectra show the difference between two redox states of *Pli*AcrIIC1. The spectrum for the reduced monomeric state is shown in red, and the one for the oxidized oligomeric state is shown in black. Source data are provided as a Source Data file.

Supplementary Table 1: NMR structure statistics for the reduced *Nme*AcrIIC1.

**NMR constraints**	
**Distance constraints**	
Total NOEs	2271
Intra-residue	448
Sequential (|i-j|=1)	562
Medium range (1<|i-j|<5)	376
Long range (|i-j|≥5)	885
Hydrogen-bond constraints	0
Torsion angle constraints	92
**Structure statistics (20 structures of lowest energy)**	
Violations	
NOE violations > 0.3 Å	0
Torsion angle violation > 5°	0
Ramachandran plot statistics	
Residues in most favored regions	84.2%
Residues in additional allowed regions	15.8%
Residues in generously allowed regions	0.0%
Residues in disallowed regions	0.0%
RMS deviations from the mean structure (Å)	
Backbone	0.44
Heavy atoms	0.75

Supplementary Table 2: X-ray crystallographic data collection and refinement statistics for the oxidized *Nme*AcrIIC1.

**Wavelength**	0.97915 Å
**Resolution range**	27.57 - 1.61 (1.668 - 1.61)
**Space group**	P 42
**Unit cell**	82.71 82.71 34.422 90 90 90
**Total reflections**	247329 (24641)
**Unique reflections**	30571 (3010)
**Multiplicity**	8.1 (8.2)
**Completeness (%)**	99.91 (99.90)
**Mean I/sigma(I)**	17.39 (2.38)
**Wilson B-factor**	26.31
**R-merge**	0.07345 (0.9489)
**R-meas**	0.07847 (1.013)
**R-pim**	0.02727 (0.3526)
**CC1/2**	0.998 (0.803)
**CC***	1 (0.944)
**Reflections used in refinement**	30565 (3010)
**Reflections used for R-free**	1465 (152)
**R-work**	0.1972 (0.2377)
**R-free**	0.2093 (0.2576)
**CC(work)**	0.966 (0.869)
**CC(free)**	0.948 (0.786)
**Number of non-hydrogen atoms**	1444
macromolecules	1261
ligands	5
solvent	178
**Protein residues**	166
**RMS(bonds)**	0.007
**RMS(angles)**	0.93
**Ramachandran favored (%)**	98.77
**Ramachandran allowed (%)**	1.23
**Ramachandran outliers (%)**	0.00
**Rotamer outliers (%)**	0.85
**Clashscore**	2.89
**Average B-factor**	30.80
macromolecules	29.36
ligands	33.06
solvent	40.94

Statistics for the highest-resolution shell are shown in parentheses.

Supplementary Table 3: Reducing buffer conditions used in previous AcrIIC1 studies *in vitro*.

Publications	Protein purification	In vitro DNA cleavage assay	Binding assay with Cas9
Mathony, J. et al. Computational design of anti-CRISPR proteins with improved inhibition potency. Nat. Chem. Biol. 16,725-730(2020).	——	20 mM Tris, pH 7.5, 100 mM KCl, 5 mM $MgCl_2$,, 5% glycerol, 1 mM DTT	——
Kim, Y. et al. Anti-CRISPR AcrIIC3 discriminates between Cas9 orthologs via targeting the variable surface of the HNH nuclease domain. FEBS J. 286, 4661-4674(2019).	——	20 mM sodium phosphate, pH 6.5, 50 mM NaCl, 1 mM PMSF 2 mM BME	20 mM sodium phosphate, pH 7.4, 100 mM NaCl, 5 mM BME
Zhu, Y. et al. Diverse Mechanisms of CRISPR-Cas9 Inhibition by Type IIC Anti-CRISPR Proteins. Mol. Cell 74, 296-309.e7(2019).	20 mM Tris-HCl, pH 7.5, 300 mM NaCl, 2 mM $MgCl_2$, 1 mM DTT	20 mM Tris-HCl, pH 7.5, 100 mM KCl, 5 mM $MgCl_{2,}$ 5% glycerol, 5 mM DTT	20 mM Tris-HCl, pH 7.5, 300 mM NaCl, 2 mM $MgCl_{2,}$ 1 mM DTT
Phaneuf, CR. et al. Ultrasensitive Multi-Species Detection of CRISPR-Cas9 by a Portable Centrifugal Microfluidic Platform. Anal Methods. 11, 559-565(2019).	50 mM Tris–HCl, pH 7.5, 500 mM NaCl, 10% glycerol, 0.5 mM TCEP	——	——
Edraki, A. et al. A Compact, High-Accuracy Cas9 with a Dinucleotide PAM for In Vivo Genome Editing. Mol Cell. 73, 714-726.e4(2019).	50 mM Tris-HCl, pH 7.5, 500 mM NaCl, 5 mM imidazole, 1 mM DTT	——	——
Seamon, KJ. et al. Versatile High-Throughput Fluorescence Assay for Monitoring Cas9 Activity. Anal Chem. 90, 6913-6921(2018).	50 mM Tris-HCl, pH 7.5, 100 mM NaCl, 0.5 mM TCEP	——	——
Rousseau, BA. et al. Programmable RNA Cleavage and Recognition by a Natural CRISPR-Cas9 System from Neisseria meningitidis. Mol Cell. 69,906-914.e4(2018).	20 mM HEPES, pH 7.5, 300 mM NaCl, 0.5 mM DTT	20 mM HEPES, pH 7.5, 50 mM KCl, 0.1 mM EDTA, 10 mM $MgCl_2$, 0.5 mM DTT	——
Harrington, L. B. et al. A Broad-Spectrum Inhibitor of CRISPR-Cas9. Cell 170, 1224-1233.e15 (2017).	50 mM Tris-HCl, pH 7.5, 20 mM imidazole, 500 mM NaCl, 1 mM PMSF, 0.5 mM TCEP-NaOH	20 mM Tris-HCl, pH 7.5, 100 mM KCl, 5 mM $MgCl_2$, 5% glycerol, 1 mM DTT	20 mM HEPES-NaOH, pH 7.5, 150 mM NaCl , 1 mM TCEP-NaOH
Pawluk, A. et al. Naturally Occurring Off-Switches for CRISPR-Cas9. Cell 167, 1829-1838.e9(2016).	10 mM Tris, pH 7.5, 250mM NaCl, 5mM BME	20 mM HEPES-KOH, pH 7.5, 150 mM KCl, 10% glycerol, 10 mM $MgCl_2$, 1 mM DTT	——

Supplementary Table 4: Redox potentials measured for the reducing buffers.

Reaction buffers	Redox potential vs. Ag/AgCl (mV)	Calibrated potential vs. SHE (Eh) (mV)
20 mM HEPES, 100 mM KCl, 10 mM $MgCl_2$, and 5% (w/v) glycerol pH 7.5, 5 mM DTT	-560 ± 34	-360 ± 34
20 mM Tris, 100 mM NaCl, pH 7.4 , 1 mM DTT	-400 ± 38	-200 ± 38
