# Supplementary material for: A redox switch regulates the assembly and anti-CRISPR activity of AcrIIC1
Source: Nat Commun. 2022 Nov 18;13:7071. doi: 10.1038/s41467-022-34551-8 (PMC9674691; doi:10.1038/s41467-022-34551-8)
Supplement: Supplementary file 3 — Reporting Summary [file 41467_2022_34551_MOESM3_ESM.pdf]

nature portfolio

Corresponding author(s): Fan Yang

Last updated by author(s): Oct 18, 2022

Reporting Summary

Nature Portfolio wishes to improve the reproducibility of the work that we publish. This form provides structure for consistency and transparency in reporting. For further information on Nature Portfolio policies, see our Editorial Policies and the Editorial Policy Checklist.

Statistics

For all statistical analyses, confirm that the following items are present in the figure legend, table legend, main text, or Methods section.

n/a	Confirmed	
☐	☒	The exact sample size (n) for each experimental group/condition, given as a discrete number and unit of measurement
☐	☒	A statement on whether measurements were taken from distinct samples or whether the same sample was measured repeatedly
☐	☒	The statistical test(s) used AND whether they are one- or two-sided<br>*Only common tests should be described solely by name; describe more complex techniques in the Methods section.*
☒	☐	A description of all covariates tested
☒	☐	A description of any assumptions or corrections, such as tests of normality and adjustment for multiple comparisons
☐	☒	A full description of the statistical parameters including central tendency (e.g. means) or other basic estimates (e.g. regression coefficient) AND variation (e.g. standard deviation) or associated estimates of uncertainty (e.g. confidence intervals)
☐	☒	For null hypothesis testing, the test statistic (e.g. F, t, r) with confidence intervals, effect sizes, degrees of freedom and P value noted<br>*Give P values as exact values whenever suitable.*
☒	☐	For Bayesian analysis, information on the choice of priors and Markov chain Monte Carlo settings
☒	☐	For hierarchical and complex designs, identification of the appropriate level for tests and full reporting of outcomes
☒	☐	Estimates of effect sizes (e.g. Cohen's d, Pearson's r), indicating how they were calculated

Our web collection on statistics for biologists contains articles on many of the points above.

Software and code

Policy information about availability of computer code

Data collection	Topspin 3.0 (For NMR data collection) , UNICORN 5.11 (For AKTA purification system), Octet Data Acquisition software version 9.
Data analysis	NMRPipe mac11_64, CcpNmr Analysis 2.4.2, TALOS+, CYANA 2.0, Amber 14.0, ForteBio Data Analysis software 9.0, PyMol 2.3.4, AlphaFold v2.0.1, Rosetta InterfaceAnalyzer (https://www.rosettacommons.org/docs/latest/application_documentation/analysis/interface-analyzer), XDS 20210323, Coot 0.9.6, autoPROC version 1.0.5, PHENIX v1.19.2_4158, GraphPad Prism 8, MEGA v7.0.26, ESPript 3.0.

For manuscripts utilizing custom algorithms or software that are central to the research but not yet described in published literature, software must be made available to editors and reviewers. We strongly encourage code deposition in a community repository (e.g. GitHub). See the Nature Portfolio guidelines for submitting code & software for further information.

Data

Policy information about availability of data

All manuscripts must include a data availability statement. This statement should provide the following information, where applicable:

- Accession codes, unique identifiers, or web links for publicly available datasets
- A description of any restrictions on data availability
- For clinical datasets or third party data, please ensure that the statement adheres to our policy

All data generated or analyzed in this study are included in the main text or the supplementary materials. Atomic coordinates and NMR data for the reduced NmeAcrIIC1 have been deposited in the Protein Data Bank (PDB) under entry ID 7X31 [https://doi.org/10.2210/pdb7X31/pdb] and Biological Magnetic Resonance Bank (BMRB) under entry ID 36471 [https://dx.doi.org/10.13018/BMR36471]. Atomic coordinates and structure factors for the crystal structures of the oxidized state of NmeAcrIIC1 have been deposited in the PDB under entry ID 7X4B [https://doi.org/10.2210/pdb7X4B/pdb]. One other published PDB code cited in this paper

is 5VGB [https://doi.org/10.2210/pdb5vgb/pdb]. The coordinates for the structural models generated in this study are provided as supporting information. The source data are provided as a Source Date file with this paper.

Field-specific reporting

Please select the one below that is the best fit for your research. If you are not sure, read the appropriate sections before making your selection.

☒ Life sciences ☐ Behavioural & social sciences ☐ Ecological, evolutionary & environmental sciences

For a reference copy of the document with all sections, see nature.com/documents/nr-reporting-summary-flat.pdf

Life sciences study design

All studies must disclose on these points even when the disclosure is negative.

Sample size	For structural calculation of the reduced NmeAcrIIC1, 100 starting structures were initially calculated and 20 structures with the lowest Amber energy were reported. The sample size was chosen based on previous similar studies. The RMSD of the 20 structures for the backbone of the secondary structural region is only 0.19 Å, indicating the sample size is sufficient. For all functional assays, three independent experiments (n=3) were performed.
Data exclusions	No data were excluded.
Replication	All the assays were replicated more than three times with similar results.
Randomization	Randomization is not relevant to our study, because there is no specific grouping in data collection or analysis.
Blinding	Blinding is not relevant to our study, because there is no specific grouping in data collection or analysis.

Reporting for specific materials, systems and methods

We require information from authors about some types of materials, experimental systems and methods used in many studies. Here, indicate whether each material, system or method listed is relevant to your study. If you are not sure if a list item applies to your research, read the appropriate section before selecting a response.

Materials & experimental systems

n/a	Involved in the study
☒	☐ Antibodies
☒	☐ Eukaryotic cell lines
☒	☐ Palaeontology and archaeology
☒	☐ Animals and other organisms
☒	☐ Human research participants
☒	☐ Clinical data
☒	☐ Dual use research of concern

Methods

n/a	Involved in the study
☒	☐ ChIP-seq
☒	☐ Flow cytometry
☒	☐ MRI-based neuroimaging
